# Dual host-pathogen small RNA sequencing during wheat stem rust infection

**DOI:** 10.1186/s13104-023-06426-8

**Published:** 2023-08-14

**Authors:** Nicholas A. Mueth, Scot H. Hulbert

**Affiliations:** 1 Molecular Plant Sciences Program, Washington State University, Pullman, Washington, USA; 2 Department of Plant Pathology, Washington State University, Pullman, Washington, USA; 3 Genome Sciences Department, University of Washington, Seattle, WA USA

**Keywords:** Wheat, Fungi, Stem rust, Plant pathogen, Small RNA, Puccinia

## Abstract

**Objectives:**

RNA sequencing of two organisms in a symbiotic interaction can yield insights that are not found in samples from each organism alone. We present a sequencing dataset focusing on the small RNA fraction from wheat plants (*Triticum aestivum*) infected with the biotrophic pathogen wheat stem rust fungus (*Puccinia graminis* f.sp. *tritici*). Simultaneous small RNA sequencing of this agronomically important crop and its adversary can lead to a better understanding of the role of noncoding RNAs in both plant and fungal biology.

**Data description:**

Small RNA libraries were constructed from infected and mock-infected plant tissue and sequenced on the Ion Torrent platform. Quality control was performed to ensure sample and data integrity. Using this dataset, researchers can employ previously established methods to map subsets of reads exclusively to each organism’s genome. Subsequent analyses can be undertaken to discover microRNAs, predict small RNA targets, and generate hypotheses for further laboratory experiments.

## Objectives

Small RNAs play an important role in plant pathology by regulating the plant immune response, and many pathogens carry small RNAs that regulate pathogenesis-related genes [1]. Additionally, small RNAs contribute to virulence directly via host-induced gene silencing and cross-kingdom RNA interference [2]. Dual host-microbe transcriptomics captures the dynamic interaction between both organisms, which is especially useful for obligate biotrophic pathogens [3]. Bread wheat (*Triticum aestivum*) expresses disease-associated microRNAs when infected with the stem rust fungus *Puccinia graminis* f.sp. *tritici* [4]. The fungus also expresses functionally distinct waves of small RNA molecules during early and late-stage infection [5]. Our lab undertook work on small RNAs in the closely-related wheat stripe rust pathogen *Puccinia striiformis* f.sp. *tritici* [6, 7]. We produced additional small RNA datasets from other *Puccinia* species with the intention of performing a comparative analysis of small RNA-producing loci across multiple rust genomes, but time and funding constraints led us to focus on *P. striiformis*. This article presents a previously unpublished small RNA sequencing dataset from the related and economically impactful plant pathogen *P. graminis*. Reads can be readily mapped to the current wheat and stem rust draft genomes available through resources such as Ensembl [8, 9]. Previously published bioinformatic methods detail how to map, filter, and partition reads into subsets corresponding to each organism [10]. Small RNA-producing loci (microRNA, tasiRNA, etc.) can be characterized using software such as ShortStack [11]. These data can then be combined with transcriptomic, RNA degradome, and/or proteomic data to associate the presence of specific small RNA sequences with the expression of protein-coding genes of interest.

## Data description

Fourteen-day old wheat plants (cultivar ‘McNair 701’) were inoculated with stem rust spores (strain Pgt7A), or mock-inoculated as an uninfected control. Infected and uninfected control treatments had three biological replicates each. Detailed methods are linked in Table 1 [12]. At four days post-infection, shoots were harvested and total RNA was extracted. Endpoint RT-PCR confirmed the presence of plant *Glyceraldehyde-3-phosphate dehydrogenase* (*GAPDH*) transcripts in all samples, and the presence of fungal *Actin* transcripts in infected reps and its absence in uninfected reps. Small RNA (< 200 nt) was isolated and confirmed via Fragment Analyzer. Adapters were added by ligation; libraries were reverse transcribed, amplified, and size-verified. Sequencing was performed on the Ion Torrent PI platform. Raw data were processed by trimming off adapters and filtering out low-quality reads (Phred < 10). FastQC was used to check read quality and generate summary statistics (Table 1).

Twenty million total reads were obtained in the sequencing run, with an average of 3.3 million reads per replicate and an average Phred quality score of 25 [13–18]. Infected and uninfected treatments had similar numbers and quality of reads. The mean read length was 24; over 75% of reads were 20–29 nt long, indicating successful selection for small RNAs.

**Table 1:** Overview of data files/data sets

Label	Name of data file	File type (extension)	Data repository and identifier
Data file 1	Uninfected Rep 1	FASTQ (.fastq)	NCBI Sequence Read Archive (https://identifiers.org/ncbi/insdc.sra:SRR24282678) [18]
Data file 2	Uninfected Rep 2	FASTQ (.fastq)	NCBI Sequence Read Archive (https://identifiers.org/ncbi/insdc.sra:SRR24282677) [17]
Data file 3	Uninfected Rep 3	FASTQ (.fastq)	NCBI Sequence Read Archive (https://identifiers.org/ncbi/insdc.sra:SRR24282676) [16]
Data file 4	Infected Rep 1	FASTQ (.fastq)	NCBI Sequence Read Archive (https://identifiers.org/ncbi/insdc.sra:SRR24282675) [15]
Data file 5	Infected Rep 2	FASTQ (.fastq)	NCBI Sequence Read Archive (https://identifiers.org/ncbi/insdc.sra:SRR24282674) [14]
Data file 6	Infected Rep 3	FASTQ (.fastq)	NCBI Sequence Read Archive (https://identifiers.org/ncbi/insdc.sra:SRR24282673) [13]
Data file 7	Methods	PDF (.pdf)	figshare (10.6084/m9.figshare.22767485) [12]
Data file 8	sRNA_FragmentAnalyzer	PDF (.pdf)	figshare (10.6084/m9.figshare.22767485) [12]
Data file 9	RT-PCR_Actin	PDF (.pdf)	figshare (10.6084/m9.figshare.22767485) [12]
Data file 10	SeqLibraries_FragmentAnalyzer	PDF (.pdf)	figshare (10.6084/m9.figshare.22767485) [12]
Data file 11	IonTorrent Run Report	PDF (.pdf)	figshare (10.6084/m9.figshare.22767485) [12]
Data file 12	FASTQC_Tae-Pgt sRNA_merged	PDF (.pdf)	figshare (10.6084/m9.figshare.22767485) [12]

## Limitations

This dataset is limited by the relatively low read depth achieved by the sequencing run. Infected wheat sequencing libraries typically have a majority of reads mapping to the host; less than 10% of reads map exclusively to the fungal genome. Despite this limitation, the dataset can provide an overview of the most abundant plant and fungal small RNAs present in this pathosystem. However, less abundant sequences may not be detected, and comparative statistics between treatments will be low-powered. This dataset can be compared with more comprehensive studies of small RNAs from stem rust [5].

## Data Availability

The data described in this Data Note can be freely and openly accessed at the NCBI Sequence Read Archive under BioProject PRJNA960906: “Small RNA sequencing of wheat infected with stem rust.” The materials described in this Data Note can be accessed in this figshare repository: 10.6084/m9.figshare.22767485.

## Abbreviations

RNA: Ribonucleic acid
sRNA: Small RNA
*Pgt*: *Puccinia graminis* f. sp. *tritici*
*GAPDH*: *Glyceraldehyde-3-phosphate dehydrogenase* gene
tasiRNA: *Trans-*acting small interfering RNA
RT-PCR: Reverse transcription followed by polymerase chain reaction
